# Exploring the Limits for Reduction of Plastid Genomes: A Case Study of the Mycoheterotrophic Orchids *Epipogium aphyllum* and *Epipogium roseum*

**DOI:** 10.1093/gbe/evv019

**Published:** 2015-01-28

**Authors:** Mikhail I. Schelkunov, Viktoria Yu Shtratnikova, Maxim S. Nuraliev, Marc-Andre Selosse, Aleksey A. Penin, Maria D. Logacheva

**Affiliations:** ^1^M. V. Lomonosov Moscow State University, Moscow, Russia; ^2^Joint Russian–Vietnamese Tropical Scientific and Technological Center, Cau Giay, Hanoi, Vietnam; ^3^Département Systématique et Evolution, Muséum National d’Histoire Naturelle, Paris, France; ^4^Kazan Federal University, Kazan, Russia

**Keywords:** plastid genome, nonphotosynthetic plants, genome reduction, gene loss, orchids

## Abstract

The question on the patterns and limits of reduction of plastid genomes in nonphotosynthetic plants and the reasons of their conservation is one of the intriguing topics in plant genome evolution. Here, we report sequencing and analysis of plastid genome in nonphotosynthetic orchids *Epipogium aphyllum* and *Epipogium roseum*, which, with sizes of 31 and 19 kbp, respectively, represent the smallest plastid genomes characterized by now. Besides drastic reduction, which is expected, we found several unusual features of these “minimal” plastomes: Multiple rearrangements, highly biased nucleotide composition, and unprecedentedly high substitution rate. Only 27 and 29 genes remained intact in the plastomes of *E. aphyllum* and *E. roseum*—those encoding ribosomal components, transfer RNAs, and three additional housekeeping genes (*infA*, *clpP*, and *accD*). We found no signs of relaxed selection acting on these genes. We hypothesize that the main reason for retention of plastid genomes in *Epipogium* is the necessity to translate messenger RNAs (mRNAs) of accD and/or clpP proteins which are essential for cell metabolism. However, these genes are absent in plastomes of several plant species; their absence is compensated by the presence of a functional copy arisen by gene transfer from plastid to the nuclear genome. This suggests that there is no single set of plastid-encoded essential genes, but rather different sets for different species and that the retention of a gene in the plastome depends on the interaction between the nucleus and plastids.

## Introduction

One of the defining characteristics of plants is their capacity to photosynthesize. However, several species have lost this ability, a phenomenon that has occurred repeatedly in the course of evolution. Such plants have adapted so that they can obtain energy from organic compounds derived from other organisms—either from other plants (parasitism; Westwood et al. 2010) or from mycorrhizal fungi colonizing their roots (mycoheterotrophy; Hynson et al. 2013). Heterotrophy is not confined to any specific lineage of plants and occurs also in bryophytes and gymnosperms, although it is more common in flowering plants, where about 1% of species, representing different lineages of dicots and monocots, are heterotrophic. Plant heterotrophy is especially interesting in the context of evolutionary biology as it represents an example of convergent evolution, a term that refers to the emergence of the same trait in distant lineages.

Plant heterotrophy is commonly associated with distinct anatomical, physiological, and genomic features. One of these is a reduction in the size of the plastid genome, or plastome, which occurs when genes encoding proteins involved in photosynthesis become unnecessary and are lost or pseudogenized (Wolfe et al. 1992). The plastomes of most photosynthetic plants are known to be highly similar in gene content and organization, while information regarding nonphotosynthetic plant plastomes is only beginning to accumulate. Earlier reports suggested conservation of plastome gene order and presence of a large set of conserved genes (Wolfe et al. 1992; Wickett et al. 2008); however, recently, highly reduced (Delannoy et al. 2011; Wicke et al. 2013) and highly rearranged (Logacheva et al. 2014) plastomes have been identified. These findings suggest that plastomes of nonphotosynthetic plants may be much more diverse than previously thought. Consistent with this idea, it was recently reported that a nonphotosynthetic algae, *Polytomella*, completely lacks a plastid genome, even though it has a plastid. When genome and transcriptome sequencing were performed, the genome was found to not contain the genes required for plastid division, DNA replication, and repair, while the genes encoding plastid-targeted proteins involved in plastid metabolism were present and expressed (Smith and Lee 2014). As an example from higher plants, possible loss of the plastid genome in the parasitic plant *Rafflesia* was reported (Molina et al. 2014), although this may be explained by a dramatic reduction in plastid DNA copy number and gene content. Taken together, these examples highlight a fact that the patterns of nonphotosynthetic plant plastome evolution are still poorly understood and additional plastome sequences representing substantially different groups of plants are needed to resolve many evolutionary questions. Importantly, among heterotrophic plants, the plastomes of mycoheterotrophs are even less well characterized than those of parasitic plants.

**Fig. 1.—:**
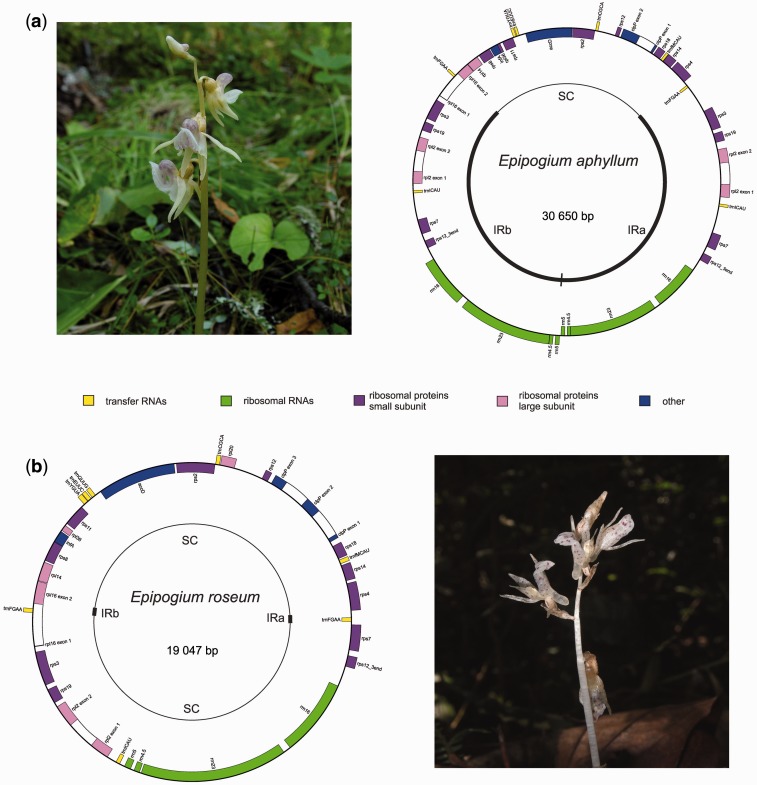
General view and plastid genome map of *Epipogium aphyllum* (*a*) and *Epipogium roseum* (*b*). Genes shown inside the circle are transcribed clockwise and those outside the circle are transcribed counterclockwise. Genes are color coded according to their functions. Photo credits: E. A. Zvyagina (*E. aphyllum*) and M. S. Nuraliev (*E. roseum*).

As a source of previously unknown plastid genome variants, the monocot family Orchidaceae is particularly attractive as it is highly diverse and includes at least 30 independent transitions to mycoheterotrophy, including some that are very ancient (Freudenstein and Barrett 2010). In this study, we characterized the plastomes of two mycoheterotrophic orchid species, *Epipogium aphyllum* and *Epipogium roseum*, which are both fully mycoheterotrophic and associated with basidiomycete fungi, but exhibit two ecologically divergent mycoheterotrophic strategies. *Epipogium aphyllum* is mycorrhizal with *Inocybe* spp., which are themselves mycorrhizal on surrounding trees, the ultimate carbon source of the plant (Roy et al. 2009; Liebel and Gebauer 2011). In contrast, *E. roseum* is mycorrhizal with saprotrophic Coprinaceae, which recover carbon from soil litter (Yamato et al. 2005; Selosse et al. 2010). *Epipogium* species are often called “ghost orchids” because of their rarity and yellow-whitish, almost transparent color (fig. 1) and the genus is quite small, consisting only of mycoheterotrophic species. Its relationship with other orchids remains uncertain, but it is presently placed into a separate subtribe, Epipogiinae, which has been thought to be a part of an exclusively mycoheterotrophic tribe Gastrodieae (Dressler 1993), although it has also been claimed that *Epipogium* is closer to the photosynthetic genus *Nervilia* (Molvray et al. 2000). Because plastid genes are commonly used and important phylogenetic markers, there have been several attempts to amplify plastid genes from *Epipogium*, all of which have failed (Cameron 2004). This led to a hypothesis that *Epipogium* has a highly divergent and reduced plastome, or that it may even have lost its plastome. To address this question, we performed low-coverage genome sequencing (genome skimming) of two *Epipogium* species, *E. aphyllum* and *E. roseum*.

## Materials and Methods

### DNA Extraction and Sequencing

Two accessions of *E. aphyllum* and five accessions of *E. roseum* were sampled (collection information is provided in supplementary table S1, Supplementary Material online). Total DNA was extracted using a modified cetyltrimethylammonium bromide (CTAB) method (Doyle 1987). Libraries were prepared using a TruSeq DNA sample prep kit v.2 (Illumina). After post-PCR purification on agarose gel, libraries were quantified using both a Qubit fluorimeter and qPCR before paired-end sequencing using either a MiSeq or a HiSeq 2000 sequencer (Illumina). Library lengths and sequencing parameters are listed in supplementary table S2, Supplementary Material online. For the White Sea accession of *E. aphyllum,* we generated a long insert (mate pair) library using Nextera Mate Pair sample preparation kit (Illumina) in addition to a standard shotgun library.

Transcriptome libraries were constructed from rRNA-depleted (Ribo-Zero Plant Leaf rRNA Removal Kit; Epicentre) total RNA using a TruSeq mRNA stranded sample preparation kit (Illumina) and sequenced using a MiSeq instrument, which yielded read lengths of 259 + 259. The samples used for transcriptome sequencing were *E. aphyllum* White Sea and *E. roseum* Vietnam 2.

### Read Preprocessing

Prior to assembly, pair-end reads were trimmed in order to remove adapters and low-quality ends using Trimmomatic 0.32 (Bolger et al. 2014). Reads were trimmed with minimal phred quality 3 from the 3′ end and with a sliding window size 5 and minimal average quality 10. Reads shorter than 50 bp were discarded. Overlapping MiSeq reads were concatenated using the fastq-join from the ea-utils toolset (http://code.google.com/p/ea-utils, last accessed April 6, 2015). The read sets were then edited to remove low-frequency k-mers using Kmernator 1.2 (https://github.com/JGI-Bioinformatics/Kmernator, last accessed April 6, 2015). This operation allowed the removal of most of the reads of nuclear origin, which constituted about 95% of all reads, and the trimming of reads containing errors. After removal of low-frequency k-mers, the plastome assembly was typically more rapid, with lower memory consumption and fewer errors. We removed k-mers of length 31 that were present fewer than 3 times. As an additional step of mate-pair read preprocessing, we also removed improperly oriented (i.e., in a forward–reverse direction) pairs using NextClip 0.8 (Leggett 2014).

### De Novo Assembly

Paired-end reads generated with the HiSeq2000 were assembled using Velvet 1.2.10 (Zerbino and Birney 2008), CLC Genomics Workbench 6.0 (www.clcbio.com, last accessed April 6, 2015), and Spades 2.5.1 (Nurk et al. 2013). With the Velvet analysis, we typically considered all k-mers from 41 to 81 with step 5 and each k-mer genomes were assembled with all values of expected coverage from 10 to 2,000 multiplying at each step by 1.5. Coverage cutoff was set at each step to one-tenth of the expected coverage. With each set of Velvet parameters, assemblies were made both with and without scaffolding. Spades was run with default parameters except additional –careful parameter. CLC Genomics Workbench was used with all default parameters and both with and without scaffolding.

To assemble the plastomes sequenced using the MiSeq, which generates longer reads than HiSeq 2000, CLC Genomics Workbench 6.0 and Newbler 2.6 (http://454.com/products/analysis-software, last accessed April 6, 2015) were used. CLC was run with default parameters, while for Newbler assemblies were made with minimum lengths of the overlapping region 100, 200, 300, 400, identity of overlapping fragments 95%, 98%, 100%, and also with default values. For the *E. aphyllum* sample White Sea, which was sequenced using both the HiSeq 2000 and MiSeq, independent assemblies were generated from the read sets.

The resulting assemblies were searched for contigs and scaffolds of plastid origin. This was accomplished by aligning plastid gene sequences of the orchids *Oncidium* Gower Ramsey, *Phalaenopsis aphrodite*, *Rhizanthella gardneri,* and *Neottia nidus-avis* to contig and scaffold sets using BLAST 2.2.26 (Altschul et al. 1990), with a BLASTN e-value 10^−^^10^ and word size 9 nt. A comparison of the plastid contig sets allowed the selection of the best contig set for further analysis. Contigs were joined using Mapsambler 1.3.17 (Peterlongo and Chikhi 2012), which extends the ends of contigs in a graph-like mode, allowing the user to investigate possible extensions. After producing putative plastome assemblies, reads were mapped (read sets prior to reduction by Kmernator) on them in order to verify their quality, using CLC Assembly Cell 4.2 (www.clcbio.com) requiring 80% of the read to align with at least 98% identity, in a mode of mapping to circular sequences (–lengthfraction 0.8 –similarity 0.98 -z). Reads were mapped to the plastome represented in two orientations: The main orientation and another orientation with 10 kbp moved from the start to the end of the sequence. The second mapping allowed us to verify the circularization of plastid genome. As an additional confirmation of the *E. aphyllum* plastome organization, reads of the mate-pair library with an insert size of 10 kb were mapped using the CLC assembly cell with the same parameters as described above (supplementary fig. S1, Supplementary Material online).

### Plastome Annotation

The assembled plastome sequences were annotated using the online tool DOGMA (Wyman et al. 2004). Taking into account the high degree of divergence between the plastomes of *Epipogium* and photosynthetic species, we used a sensitive set of parameters: e-value of a match to reference genes 10^−^^5^ and match identity 25% (minimal allowed value). Gene predictions suggested by DOGMA were manually checked. The validity of the DOGMA predictions was further assessed by aligning *Oncidium* Gower Ramsey, *P. aphrodite*, *R. gardneri,* and *N. nidus-avis* genes using BLASTN and TBLASTN. To generate more precise transfer RNA (tRNA) gene predictions, we scanned the whole plastomes using tRNAscan-SE (Lowe and Eddy 1997) with the mito/chloroplast prediction model at http://selab.janelia.org/tRNAscan-SE (last accessed April 6, 2015). Finally, we made a multiple comparison of *Epipogium* plastomes using mVista (Frazer et al. 2004), a web server for comparative genome analysis, to identify highly conserved but unannotated regions, presumably corresponding to unrecognized genes.

### Analysis of Plastomes

To determine gene sequence identity, we performed multiple alignments of concatenated sets of common genes or proteins using MUSCLE 3.8.31 (Edgar 2004) (for RNA-coding genes and protein sequences) and TranslatorX+MUSCLE (for nucleotide sequences of protein-coding genes), and BioEdit 7.2.5 (Hall 1999) was used to build identity tables from the alignments. Insertions and deletions were counted using an in-house script.

A search of repeats in the plastomes was performed using Vmatch 2.2.1 (http://www.vmatch.de, last accessed April 6, 2015). Prior to the search, we removed one copy of inverted repeat (IR) from all plastomes under consideration. The requirements for repeat detection were as follows: Repeats should be no less then 20 bp in length, identity between the copies of the repeat should be no less than 90%, edit distance between them should be no more than 10 (the edit distance is the sum of numbers of mismatches and gaps). An overlap between two units of a repeat pair was not allowed and searches were made for both direct repeat and IR. There were no restrictions on maximum distance between repeat units.

The tree topology was taken from Górniak et al. (2010), where it was inferred from nuclear *Xdh* (xanthine dehydrogenase) gene sequences. The topology of the *Epipogium* subtree was inferred from sequences of nuclear 18S rRNA gene sequences found among de novo assembled contigs using BLAST. Sequences of 18S rRNA gene of *Epipogium* samples and *N. nidus-avis*, used as outgroup, were aligned using MUSCLE 3.8.31 (Edgar 2004) with default parameters. A tree was built using RAxML 8.2 (Stamatakis 2014) with a GTR+GAMMA model, with verification using 1,000 fast bootstrap replicates. The resulting topology was nested into the general orchid tree build by a maximum parsimony method by Górniak et al. (2010). Because *Epipogium* sequences were not used in that study, we extrapolated its position from that of its putative relatives, species from tribes Nervilieae, Gastrodieae, and Triphorae (Molvray et al. 2000; Rothacker 2007). All of them are basal braches of epidendroid orchids (Górniak et al. 2010). To add branch lengths, we used 14 protein-coding genes that are common to all orchids with a characterized plastome (*rpl2*, *rpl14*, *rpl16*, *rpl36*, *rps2*, *rps3*, *rps4*, *rps7*, *rps8*, *rps11*, *rps14*, *rps19*, *accD*, *clpP*). Nucleotide sequences were aligned using the respective amino acid sequences, using TranslatorX 1.1 (Abascal et al. 2010), which utilizes MUSCLE. PartitionFinder 1.1.1 (Lanfear et al. 2014) was then used to estimate an optimal separation of gene sequences into sets with different GTR + GAMMA model parameters. PartitionFinder combined the 42 input sets (14 genes with 3 codon positions in each) into 15 sets based on a corrected Akaike information criterion. RAxML then added branch lengths to the known topology (“-f e” mode) using the concatenated gene sequences and partition.

All dN/dS values were calculated using PAML 4.7 (Yang 2007). To test if there is difference between dN/dS values on branches of the *Epipogium* subtree and on branches of other orchids, we conducted two independent calculations using PAML. In the first case, it was assumed that all the branches have the same dN/dS (model = 0), while in the second case two different dN/dS ratios were allowed for *Epipogium* and non-*Epipogium* branches (model = 2). The *P* value of a particular hypothesis was calculated using a likelihood ratio test. We used a branch model with an estimation of codon usage using an F3x4 model. The initial dN/dS value was set to 0.5 and the initial ratio of transitions to transversions frequency to 2.0. The analysis was conducted with a concatenated set of common orchid protein-coding genes, aligned using TranslatorX+Muscle. The tree topology was inferred as described above. Branch lengths for the tree were computed using PAML (fix_blength = 0). In addition, we made a calculation in a mode that allows each branch to have its own dN/dS value (model = 1)

For pairwise dN/dS evaluations, we aligned orchid genes separately using TranslatorX+Muscle and then performed the PAML analysis in a pairwise mode (runmode = −2). The initial dN/dS and initial transition to transversion ratios were set to 0.5 and 2.0, respectively, with a codon frequency model F3x4.

Codon usage, amino acid usage, and Guanine-Cytosine (GC) content were estimated using CodonW 1.4.2 (Peden 1999). These analyses were made for orchids *Oncidium* Gower Ramsey, *R. gardneri,* and *E. roseum* sample Vietnam 2. The statistical significances of differences in codon and amino acid usage and GC-content were estimated using a pairwise proportion Z-test, with Bonferroni correction in cases of multiple comparisons.

To compare gene sets from different species, we used annotations deposited in GenBank or kindly provided to us by Susann Wicke from the University of Vienna (for annotations of Orobanchaceae). Unfortunately, some annotations have ambiguous gene descriptions and mistakes, so the results of our calculation of gene numbers may deviate slightly from those reported in corresponding publications.

### Transcriptome Analysis

Reads from RNA sequencing were trimmed using a quality score of 3 with adapter removal using Trimmomatic. To manually confirm the presence of gene transcription, we mapped reads using CLC Assembly Cell. We used various requirements for mapping strictness, requiring a portion of a read that must map to vary from 0.3 to 1.0 and mapping identity from 80% to 100%. Stricter parameters help avoid nonspecific mapping, while less strict parameters allow reads with RNA editing and sequencing errors to be more readily mapped. To check for the presence of splicing, we artificially joined exons and mapped reads to them in a strict mode, demanding whole reads to map with 98% identity.

## Results

### Plastome Size, Structure, and Gene Content

We characterized the plastomes of five accessions of *E. roseum* and two accessions of *E. aphyllum* and determined that they are highly reduced and rearranged in both species compared with other plants, including those that are nonphotosynthetic. The plastome size of *E. aphyllum* is ∼30.5 kb and, in contrast to the other plastomes, which are either quadripartite, with two single-copy (SC) regions (one large one and one small), and an IR or lack of repeat region, it has an IR region, albeit one SC region (fig. 1). The *E. aphyllum* plastome is predicted to contain 27 unique genes, including 17 protein-coding genes (ribosomal proteins, a subunit of a chloroplast protease [*clpP*], acetyl-CoA carboxylase [*accD*], a translation initiation factor [*infA*]), 4 ribosomal RNA genes as well as 6 transfer RNAs. Two genes, *rps11* and *rps18*, have a highly divergent 5′ end and are shorter than the orthologs from other species. *Epipogium aphyllum* retains four intron-containing genes: *rpl2*, *clpP*, *rpl16,* and *rps12*. *clpP* has only one intron, while in most species the orthologous gene has two introns, although the length of coding region is similar. In *rpl2*, the intron position is conserved and the gene is highly similar to its orthologs across its whole length, including the noncoding region (81% identity in the exons and 73% in the introns). Additionally, the *rpl2* gene has an atypical start codon; one that is presumably converted to the canonical ATG codon by RNA editing, a feature commonly found in all monocots, whether photosynthetic or not. *rpl16* has a very short first exon (9 nt), which complicates its identification, and a region with high similarity to the second exon is located near the IR–SC junction. We identified the predicted first exon of *rpl16* as being located within the IR and separated from the second exon by a 914-bp region, ∼200 bp of which has moderate similarity (68%) with the *rpl16* intron of other orchids. In addition, a gene encoding trnF-GAA is found within this region and, as far as we are aware, this represents the first report of transfer RNA gene within another gene. *rps12* is a trans-splicing gene that consists of three exons, located in two different regions of plastome: The first exon is in the SC region and second and third are in the IR. In *E. aphyllum*, both parts of *rps12* have high similarity with the corresponding sequences of other species, except for the last exon, which is either absent or highly divergent. We determined that the reduction of plastome size in *E. roseum* is more substantial due to the extreme contraction of the IR region—only a small region carrying the *trnF* gene is duplicated. The gene content of the *E. roseum* plastome is similar to that of *E. aphyllum*, except that *E. roseum* has two additional genes: A transfer RNA (*trnQ*-UUG) and a protein-coding gene *rpl20.* In contrast to *E. aphyllum*, the *clpP* gene of *E. roseum* has three exons, and we were able to identify only two exons in the *E. roseum rps12* gene. Notably, in both species the fraction of coding DNA is very high (67–74%).

The plastomes of both *Epipogium* species show evidence of substantial rearrangement. For example, when compared with *Oncidium*, a photosynthetic orchid that has a typical plastome gene order, eight collinear blocks are present in *E. aphyllum* and three blocks are conserved between *E. aphyllum* and *E. roseum* (supplementary figs. S2 and S3, Supplementary Material online). Notably, despite numerous rearrangements, the genes that are known to constitute operons are conserved in terms of their order (e.g., rRNA genes, S10 operon, *clpP*-5′-*rps12*). *Epipogium aphyllum* and *E. roseum* have an almost identical gene order, except for the structure of the IR, which is highly reduced in *E. roseum*. Such a reduction could have arisen by two events: Inversion of the *rps7*–*rrn5* part of IRb and almost complete loss of IRa (from *rrn5* to *rps3*), or deletion of the *rps7*–*rrn5* part of IRb and deletion of *trnI*–CAU–*rps3*.

### Intraspecific and Interspecific Sequence Divergence

**Fig. 2.—:**
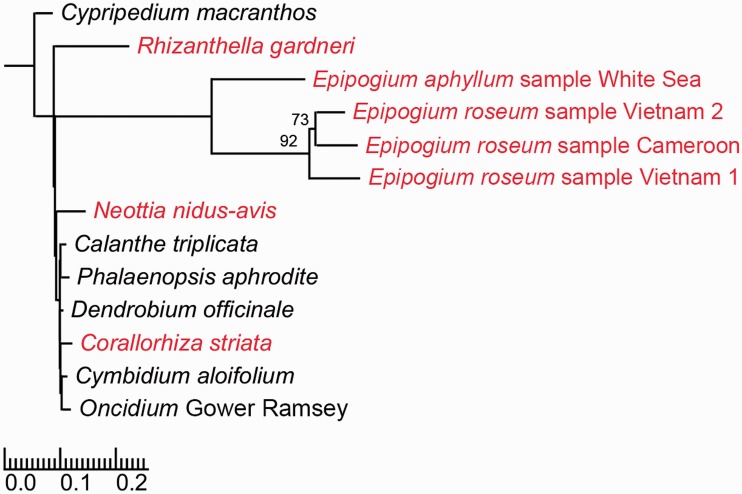
Phylogenetic tree of orchids with known plastome sequences (general tree topology is following Gorniak et al. 2010, branch lengths are inferred from the analysis of 14 shared plastid protein-coding genes). Plants that have lost the capacity for photosynthesis are labeled by red. The scale bar designates the number of substitution per nucleotide. Numbers above branches indicate bootstrap support values for the *Epipogium* subtree (inferred from the analysis of 18S rRNA gene sequences).

Sequencing of multiple individuals from the same species allowed us to assess intraspecific plastome polymorphism. In the two European *E. aphyllum* samples, sequence identity is very high (∼99%), with the differences corresponding to both single nucleotide substitutions and indels in both the noncoding and coding regions, and gene order and content are identical. In *E. roseum,* we revealed much higher diversity and the five samples analyzed from three equally distant groups. The first group includes two samples from Vietnam and a sample from Vanuatu, while the other two groups are represented by one sample each from Cameroon and Vietnam. The overall sequence similarity between these groups is 79–82% versus 98–99% within the Vietnam–Vanuatu group (supplementary table S3, Supplementary Material online). Noncoding sequences are the most polymorphic, with a prevalence of indels over single nucleotide substitutions. In the protein-coding sequences, the similarity is 0.994–0.999 within group and 0.836–0.864 between groups. rRNA genes comprise the most conserved part of the genome and their similarity within a group is 0.998 and between groups from 0.912 to 0.924. The sequence identity between *E. aphyllum* and *E. roseum* is ∼0.73–0.74 for protein-coding genes and 0.84–0.85 for rRNA genes. Among photosynthetic species, even those representing distant lineages such as *Oncidium* and *Phalaenopsis*, sequence similarity is much higher: 0.94–0.96 for protein-coding and 0.99 for rRNA genes (supplementary table S4, Supplementary Material online). An increase in substitution rate is also apparent in *Neottia* and *Rhizanthella*, although each has lost the capacity for photosynthesis independently (fig. 2). An increase in the substitution rate in plastomes of parasitic plants is a known phenomenon (Bromham et al. 2013), although to our knowledge such high divergence at an intraspecific level has not previously been reported.

Indels between different *E. roseum* groups are abundant: We identified 200–250 with average length of 7.5–7.9 bp (30–43 indels between the genomes of one group). Length mutations predominantly occur in GC-poor regions (supplementary table S5, Supplementary Material online). Differences between *E. roseum* accessions also influence IR structure and gene content. In the sample from Cameroon, the two parts of IR are not identical, with only 93% similarity, but are identical in all other *E. roseum* samples. As mentioned above, the IR in *Epipogium* harbors the *trnF* gene. In most accessions of *E. roseum* (as well as in *E. aphyllum*), this is trnF-GAA, but in the Cameroon sample a mutation affecting the anticodon is found, converting trnF-GAA into trnF-AAA. trnF-GAA is typical for plant plastomes, whereas trnF-AAA has not been reported before. Its functionality in *E. roseum* Cameroon still requires validation (see Discussion), but regardless of whether or not this is a pseudogene or a new tRNA, we observed a difference in gene content in accessions of the same species.

### GC Content

*Epipogium* plastomes have a very low GC content, with 30–31% in *E. roseum* and 32.8–32.9% in *E. aphyllum* (table 1). The distribution of nucleotides is very uneven across the genome, and the IR region in *E. aphyllum* and the homologous region in *E. roseum* are more GC rich (37%), while the SC region in *E. aphyllum* and homologous region in *E. roseum* have only 20% GC. The higher than average GC content in *E. aphyllum* is due to the fact that it has a long IR; specifically, the more GC rich part of the plastome is duplicated. In addition, the GC content differs greatly between the coding and noncoding parts of the plastome. Noncoding regions are the most GC poor: 8–14% in SC/SC-like intergenic spacers and 17–21% in IR/IR-like spacers (SC-like and IR-like refer to regions of the *E. roseum* plastome that are homologous to the IR and SC of *E. aphyllum*, respectively). In contrast, the most GC rich part of the plastome corresponds to rRNA genes (supplementary fig. S4, Supplementary Material online). In the protein-coding genes, the GC content in different positions of the codons differs, with the first positions being the most GC rich and third positions the most GC poor. The same is a general characteristic of both photosynthetic and nonphotosynthetic orchids; however, in *Epipogium,* the GC content in all three positions is significantly lower than in other orchids (*P* < 10^−^^4^). In contrast to other orchids, the difference between the GC content of the first and second positions in *Epipogium* is smaller (supplementary fig. S4, Supplementary Material online) and such convergence of GC content values is also observed in holoparasites from the Orobanchaceae (Wicke et al. 2013).

**Table 1:** Summary of *Epipogium* Plastid Genome Features

Sample	Plastome Length (bp)	GC Content (%)	Total Number of Genes^a^	Protein Coding^a^	rRNA^a^	tRNA^a^	Fraction of Coding DNA (%)^b^
*Epipogium aphyllum* sample White Sea	30,650	32.8	27	17	4	6	67
*E. aphyllum* sample France	30,594	32.9	27	17	4	6	67
*Epipogium roseum* sample Cameroon	18,339	31.0	29	18	4	7	74
*E. roseum* sample Vietnam 1	18,938	30.1	29	18	4	7	71
*E. roseum* sample Vietnam 2	19,047	30.6	29	18	4	7	73
*E. roseum* sample Vietnam 3	19,015	30.0	29	18	4	7	71
*E. roseum* sample Vanuatu	18,966	30.1	29	18	4	7	71

^a^Number of unique genes (not including copies in IR).

^b^Including RNA genes.

### Repeat Content

The fraction of repeats in the plastome of photosynthetic orchids is about 11–12%, and in *Epipogium* it ranges from 9.4 to 15.4% (supplementary table S6, Supplementary Material online). In contrast to other orchids, in *Epipogium,* repeats are extremely AT rich (92.5–94.2% AT) and most represent AT homopolymers and other low-complexity sequences. The distribution of repeats across the genome is uneven and they are much more abundant in noncoding than in coding regions, and in SC regions rather than in IR regions. The IR-like region of the *E. roseum* plastome also has a decreased density of repeats compared with a region that corresponds to the SC (supplementary fig. S5, Supplementary Material online).

### Synonymous and Nonsynonymous Substitution Rates

To evaluate the selective pressure acting on the *Epipogium* plastid genes, we first analyzed a concatenated set of genes from *Epipogium* and other orchids. This analysis indicated that the selective force acting on *Epipogium* genes does not, on average, differ significantly from that acting on the genes of other orchids (*P* = 0.44). Averaged by the branches of the *Epipogium* clade, the dN/dS is 0.21, while on the branches of other orchids it is 0.20. Despite the fact that in *Epipogium* dS is substantially greater, the dN value increases proportionally and so the dN/dS ratio remains the same (supplementary fig. S6, Supplementary Material online). Pairwise comparisons of genes of *Epipogium* and a photosynthetic orchid *Oncidium* Gower Ramsey confirmed that the dN/dS value is low (supplementary fig. S7, Supplementary Material online). This supports the idea that selection pressure on *Epipogium* plastid gene sequences is not relaxed. The ratio between the numbers of nonsynonymous to synonymous substitutions within one species, *E. roseum* (pN/pS), is higher than dN/dS, further confirming negative selection (Eyrewalker 2006) (supplementary table S7, Supplementary Material online).

### Evolution of tRNA Genes: High Sequence Divergence and Compensatory Mutations

*Epipogium* plastomes carry six (*E. aphyllum*) or seven (*E. roseum*) tRNA genes, most of which are conserved in other nonphotosynthetic plants, with the exceptions of *trnF*-GAA, which is absent from *Phelipanche ramose* (Wicke et al. 2013), and *trnC*-GCA which is absent from *Epifagus virginiana* (Wolfe et al. 1992). The gene *trnQ*-UUG is absent from *E. aphyllum*, although it is present in all nonphotosynthetic plants studied so far. The sequence similarity between tRNA genes of *Epipogium* and the photosynthetic orchid *P. aphrodite* (81–93%) is much less than usually observed for tRNAs. *Epipogium* trnY-GUA genes differ from those of *Phalaenopsis* not only with respect to substitutions but also by a 1-nt insertion. Length mutations in tRNAs are extremely rare; however, in silico tRNA folding analyses showed that most tRNAs have typical secondary structure and most positions that participate in hairpin formation are either conserved or substitutions occur in both nucleotides, such that complementarity is maintained (compensated substitutions; supplementary table S8, Supplementary Material online). The most striking observation related to the tRNA genes of *Epipogium* is the structure of *trnF* in the *E. roseum* sample from Cameroon. Although *E. aphyllum* and other *E. roseum* samples have a trnF-GAA that is typical of plant plastomes, in the Cameroon sample a substitution in the anticodon converts GAA into AAA. This sample has two slightly different *trnF* sequences (sequence similarity 93.5%), both of which have two noncompensated substitutions in the acceptor stem and low cove scores (supplementary table S8, Supplementary Material online; 40 and 23.8). (The lowest score required by tRNAscan-SE to accept a sequence as an organellar tRNA is 15).

### Evolution of Protein-Coding Genes: Codon and Amino Acid Bias

**Fig. 3.—:**
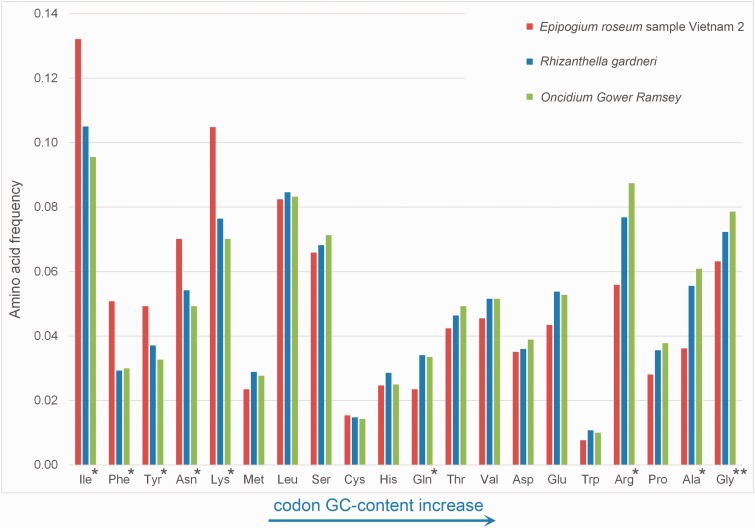
Amino acid usage in three orchids. Amino acids are listed in the order of increase of average codon GC content (e.g., isoleucine has the codons ATA, ATT, and ATC with an average GC content 1/9, and glycine has the codons GGG, GGC, GGA, and GGT with an average GC content 10/12). Single asterisk denotes cases when amino acid usage in *E. roseum* differs significantly (*P* < 0.05) from both the other orchids. Two asterisks indicate that the difference is significant only from the green orchid *Oncidium* Gower Ramsey.

*Epipogium* plastomes have higher AT content than it is usually observed in plant plastomes. The most AT-rich component is noncoding DNA, but the AT bias is also seen in protein-coding sequences and substantially changes codon usage. With respect to synonymous codons, those that are AT-rich ones have a strong predominance over GC-rich codons. Although a greater frequency of A/U-ending codons is common in most plastomes, in *Epipogium* it is significantly higher than in photosynthetic orchids. For example, for phenylalanine, which is encoded by UUU and UUC, the fraction of UUU is 65% in *Oncidium* and 91% in *Epipogium* (supplementary table S9, Supplementary Material online). Consistent with this, the effective number of codons (ENC) in *Epipogium* plastid genes is 36–40, while in photosynthetic orchids the equivalent range is 47–48. In other nonphotosynthetic orchids, ENC values remain within, or very close to, this range (supplementary table S10, Supplementary Material online). AT bias, which is a prominent feature of *Epipogium* plastomes, is apparent not only at the level of synonymous codon usage, but also at the amino acid usage level. A comparison of amino acid frequency in genes that are shared between *Epipogium* and other orchids showed that amino acids encoded by GC-poor codons, such as Phe and Ile, are significantly more frequent, and the frequency of amino acids encoded by GC-rich codons (Ala, Arg) is lower (fig. 3).

### Expression of Plastid Genes

In order to test whether plastid genes in *Epipogium* are expressed, spliced, and edited, we mapped transcriptome reads onto the annotated plastome sequences. In both species, all the genes had mapped reads, except for *rps18*. In addition, all four intron-containing genes, reads that overlap predicted exon junctions were identified (supplementary fig. S8, Supplementary Material online). RNA-seq confirmed the presence of a single intron in *E. aphyllum clpP*, which differs from the two introns present in *E. roseum* and most other plants. However, the frequency of spliced transcripts was different. In *clpP* and *rps12,* the coverage of a region of the exon junction is approximately the same as within exons, while in *rpl2* it is much lower, especially in *E. roseum*. This indicates a lower splicing rate. In addition to splicing, editing (C to U) of the second position of the *rpl2* start codon was identified, although at a low frequency (83 out of 157 reads in *E. aphyllum* and 23 reads out of 176 in *E. roseum*).

## Discussion

**Fig. 4.—:**
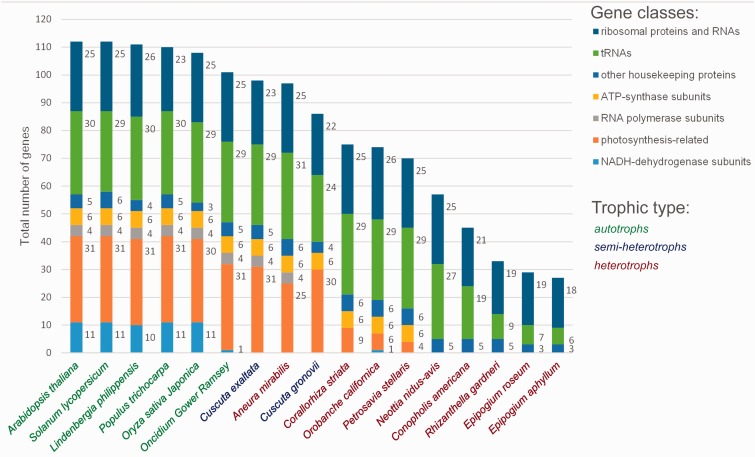
The patterns of plastid gene loss in different groups of photosynthetic and nonphotosynthetic plants. Species are ordered by a number of unique genes (not taking into account duplicated genes).

In this study, we characterized the plastomes of *E. aphyllum* and *E. roseum*, which we showed to be highly rearranged and reduced (31 and 19 kb, respectively). The latter is the smallest plastome reported to date and even the highly reduced residual plastomes of parasitic protists (such as *Plasmodium* and *Helicosporidium*) are larger, with size about 35 kb (Wilson et al. 1996; de Koning and Keeling 2006). Also, peridinin-containing dinoflagellates have plastomes in the form of several mini-circles 2–3 kb in length (Zhang et al. 1999) with a total length of 27–46 kb (Barbrook et al. 2014). The sizes of plant plastomes, including nonphotosynthetic species, range from 45.7 (*Conopholis americana*, a parasite) to 217 kb. We show here that the reduction can go further and affect ∼85% of the typical plastome size (∼150 kb) and 75% of the total gene complement. Besides heterotrophic plant species, a reduction in plastome size has also been reported in photosynthetic plants under several experimental conditions (Day and Ellis 1985; Cahoon, Cunningham, Stern 2003; Cahoon, Cunningham, Bollenbach, et al. 2003), but the patterns of reduction differ in these two cases. In experimental systems, different cell lines carry different deletions and the lines that started heterotrophic evolution at the same time have dissimilar degrees of reduction. In contrast, in heterotrophic plants, there is a clear parallel in the patterns of gene loss in lineages that transition to heterotrophy independently. This has led to a postulation of the existence of a conserved minimal gene set (Delannoy et al. 2011; Logacheva et al. 2011), to which all the reduced plastomes should converge. This core set was thought to include rRNA genes, several tRNA genes, most ribosomal protein genes, *clpP*, *accD*, *ycf1,* and *ycf2*. Later a model describing the pattern of gene loss during plastome degradation was proposed (Barrett and Davis 2012; Barrett et al. 2014). In postulates that changes in a plastome gene set of plants that lose their photosynthetic ability follow a specific path. First, the plastome loses genes encoding subunits of reduced nicotinamide adenine dinucleotide (NADH) dehydrogenase. This can be explained by the fact that this complex plays a supplementary role in photosynthesis and plants with knocked-out *ndh* genes are viable (Burrows et al. 1998). The second group of genes to be lost are those responsible for photosynthesis and the third in the sequence is the plastid-encoded RNA polymerase gene. This RNA polymerase, in contrast to the nuclear-encoded polymerase, is responsible mainly for the transcription of genes lost in the first two steps, and so it becomes unnecessary. Genes encoding transfer RNA and ribosome components and several other housekeeping genes—*matK*, *clpP*, *infA*, *accD*, *ycf1*, and *ycf2*—are the last ones. In general, the gene content of the *Epipogium* plastomes agrees with this model, being close to the end of this path (fig. 4).

Despite high degree of reduction, several lines of evidence support that *Epipogium* plastome is not just a remnant of formerly functional plastome but a functional entity. First, there is a high percentage of coding DNA; more than in other nonphotosynthetic plants (Wicke et al. 2013) and even more than in photosynthetic orchids that have lost functional *ndh* genes but retain their pseudogenes in the plastome (Chang 2005; Wu et al. 2010). Second, there is a conservation of the nonrandom gene set, an absence of a dN/dS increase in proteincoding genes, and an abundance of compensatory substitutions in tRNA genes. Third, we found expression, editing, and splicing of plastid genes in both *Epipogium* species. Regulation of these processes relies completely on nuclear gene products (in photosynthetic plants, some elements of the transcription and splicing machinery, such as RNA polymerase and the splicing factor *matK,* are plastid encoded but this is not the case for *Epipogium*) and requires complex and co-ordinated nucleus-plastid interactions. This machinery is unlikely to be conserved in the absence of function and indeed it has been lost in the plastome-less alga *Polytomella* (Smith and Lee 2014). The only presumable pseudogene in *Epipogium* plastomes is *trnF-AAA*, found in one of *E. roseum* accession. *trnF-AAA* has not been reported to be present in plant plastomes; however, it is an isoacceptor of *trnF-GAA* that is commonly found in plant plastomes, including those that are nonphotosynthetic. Mutations in anticodons (anticodon shifts) occurred many times in the evolution of several genomes, with isoacceptor shifts being more frequent than alloacceptor shifts (Rogers and Griffiths-Jones 2014). The change of *trnF-GAA* to *trnF-AAA* may have been favorable, because ∼90% of all Phe codons in *Epipogium* are encoded by UUU. However, *trnF-AAA* does not retain a secondary structure that is typical of transfer RNAs as it has two noncompensated mutations in the acceptor stem. It has been shown in multiple experiments with *Escherichia coli* and other model systems that proper pairing in the acceptor stem is crucial for tRNA recognition by aminoacyl tRNA synthetase (Jahn et al. 1991). Thus, the presence of two noncompensated mutations in the acceptor stem strongly suggests nonfunctionality. However, a similar situation has been observed in the mitochondrial genomes of several invertebrates, including *Lithobius*, where most tRNAs have mismatches in the acceptor stem that are rectified by RNA editing (Lavrov et al. 2000). The same has been shown for plant mitochondrial transfer RNA *trnF-GAA* (Maréchal-Drouard et al. 1993). Similar mechanism may occur in *E. roseum*, restoring proper pairing in *trnF-AAA*.

Our analysis indicates that *Epipogium* plastomes underwent changes not only in gene content but also in structure. Although most plastomes have a quadripartite structure, with a long identical IR separated by two SC regions, *E. aphyllum* lacks the SSC and *E. roseum* has a very short IR. The loss of the SSC is expected as it usually contains *ndh* genes that are pseudogenized in many plants, including orchids (Chang 2005), as well as several photosynthesis-related genes. Comparative sequence analysis of *E. aphyllum* and *E. roseum* clearly indicates that the structure found for *E. roseum* is more derived. The *E. roseum* plastome region that corresponds to the IR has a higher GC content and is less divergent than that corresponding to the SC. In one *E. roseum* accession (Cameroon), we identified a nonidentical IR, while in most plants studied to date, parts of the IR are identical, even in plastomes with a very small IR. This identity is thought to be maintained by gene conversion (Palmer 1985). The occurrence of a nonidentical IR suggests that this mechanism may have been lost or suppressed in *E. roseum*.

Sequencing multiple accession of *Epipogium* allowed us to assess the level of intraspecific plastome polymorphism—the first intraspecific comparison of complete plastid genome sequences from nonphotosynthetic plants. We found an unexpectedly high divergence between *E. roseum* individuals, including those sampled not very far geographically from another (Vietnam 1 and 2 are situated about 100 km from each other). Although plastid DNA, especially in noncoding regions, displays some variation in most plant species, it is more than 10 times less than we found in *E. roseum* (Xu et al. 2012). Because little is known about the population genetics of *E. roseum*, which covers a wide range of longitudes and climates, we cannot exclude the possibility that these samples represent cryptic species. However, a recent survey of polymorphism in the *atpB-rbcL* region in another nonphotosynthetic orchid, *N. nidus-avis*, revealed that it is highly divergent between individuals from the same population (Cafasso and Chinali 2012). A comparison of several plastome regions from different populations of *R. gardneri* also showed high sequence divergence (Delannoy et al. 2011) suggesting that this is common for nonphotosynthetic plants.

A high substitution rate is not usual for plant plastomes. Among all DNA-containing organelles, those with the highest rate of substitution accumulation are animal mitochondria (Lynch and Blanchard 1998). Several hypotheses have been proposed to explain this: The absence of recombination and low effective number (i.e., despite a high number of mitochondria in somatic cells, few are present in the egg cell and are transmitted to the progeny) causing mutational drift (Neiman and Taylor 2009). These explanations may be applicable to the *Epipogium* plastome as well. Increased substitution rates in all three genomes were found in parasitic plants and it has been suggested that this may reflect a shorter generation time or decreased efficiency of DNA repair (Bromham et al. 2013). High substitution rates were also observed in the 18 S nuclear rRNA gene of mycoheterotrophic species (Lemaire et al. 2011), suggesting that rate acceleration is a characteristic of the genomes of all heterotrophic plants, and not only those that are parasitic. However, no evidence of accelerated sequence evolution was found in the mycoheterotrophic monocot *Petrosavia stellaris* (Logacheva et al. 2014). More data, including mitochondrial genome sequences, are necessary in order to determine whether the extremely high substitution rates of *Epipogium* plastomes are unique or reflect general trends of genome evolution in heterotrophic plants. Interestingly, a high substitution rate and high AT content are also typical in symbiotic prokaryotes with very small genomes (McCutcheon and Moran 2011).

Summarizing our findings, characterization of *Epipogium* plastomes reveals highly dynamic evolutionary patterns that are however not “chaotic” as in heterotrophic cell cultures and that are directed by high substitution rate and negative selection.

Several hypotheses have been proposed to explain the conservation of plastid genomes in nonphotosynthetic plants (reviewed in Barbrook et al. 2006). The most plausible is the essential tRNA hypothesis, which postulates that *trnE*-UUC is the main reason for plastome conservation since it is essential for heme biosynthesis. Our analysis of the *Epipogium* plastomes does not contradict this hypothesis as in both species *trnE* is intact; however, it does not explain the retention and expression of other plastid genes. Other than photosynthesis, plastids are also involved in several processes that are crucially important even for nonphotosynthetic plants. These processes include the synthesis of fatty acids and carotenoids, starch storage and gravitropism (Chen et al. 1999; Neuhaus and Emes 2000). In model plant species, complex interactions that involve feedback loops exist between the translation of plastid genes and other processes (Tiller and Bock 2014). These interactions mainly co-ordinate photosynthesis-related processes; however, it was shown that plastid translation is also required for *Arabidopsis thaliana* embryo development (Romani et al. 2012). Several lines of evidence indicate that this phenomenon is not related to photosynthesis, but rather to the necessity of the *accD* gene in fatty acid biosynthesis (Bryant et al. 2011). *Epipogium* plastomes encode only two genes, which are involved in functions other than translation and, notably, *accD* is one of these. Another gene, *clpP*, is essential for tobacco shoot development (Kuroda and Maliga 2003; but see Cahoon, Cunningham, Stern 2003; Cahoon, Cunningham, Bollenbach, et al. 2003). We suggest that associations between translation of these plastid genes and development, similar to those reported for *A. thaliana* and tobacco, exist in *Epipogium* and that this underlies the necessity of plastome retention.

Recently, the apparent absence of a plastid genome was reported in *Rafflesia lagascae*, a parasitic dicot (Molina et al. 2014). This seems to contradict the essential tRNA hypothesis as well as our hypothesis, which can be termed the “essential plastid translation hypothesis.” However, it is well known that different plant lineages have different necessity in plastid-encoded gene products. *rpl22* and *rps16* are essential for tobacco (Fleischmann et al. 2011), while in legumes they are lost from the plastome (Doyle et al. 1995). The *accD* gene of grasses and Campanulaceae has been lost from the plastome and its product has been replaced by a plastid-targeted, but nuclear-encoded gene (Rousseau-Gueutin et al. 2013). Consistent with this, the breakdown of translation in maize plastids does not lead to embryo lethality (Asakura and Barkan 2006). The same is true for *clpP*—as mentioned above, it is essential for tobacco, but not for maize (Cahoon, Cunningham, Stern 2003; Cahoon, Cunningham, Bollenbach, et al. 2003). Even more striking, in *Brassica napus*, a close relative of *A. thaliana*, plastid translation is not necessary for embryo development (Zubko and Day 1998) because *accD* gene encoded in the *B. napus* plastome is replaced by, or at least complemented with, the plastid-targeted nuclear gene product (Schulte et al. 1997). This emphasizes that even within closely related plant groups the requirements for plastid-encoded genes can differ. If the product of a plastid gene is essential for a plant, it can be lost from the plastome when a functional copy exists in the nuclear genome. Such copies are the results of gene transfer. Gene transfer from an organelle to the nucleus is a multistep process that includes: 1) integration of the sequence into the nuclear genome; 2) its activation (acquisition of translation and transcription); 3) gain of a transit peptide; and 4) loss from the organellar genome (Selosse et al. 2001). The first step, integration, is quite common in plants, as has been shown by both comparative genome analysis and direct experimental methods (Huang et al. 2003; Shahmuradov et al. 2003). The probability of integration correlates with the number of plastids (Smith et al. 2011) and thus can be species- or lineage specific. Although a functional gene is present in the plastome, all transformations involving its nuclear counterpart occur at random and are not under selection. Thus, we should not expect that their consequences are streamlined and lead to the same results in different species. There is therefore probably not a single set of “essential genes” for all nonphotosynthetic plants, but rather different sets of essential genes for different plants, depending on the interactions between the plastid and the nucleus that have been established over the course of evolution. We believe that exploration of nuclear genes and their structure and expression in both nonphotosynthetic plants and their photosynthetic relatives (including our forthcoming study on *Epipogium* transcriptomes) will provide new insights into these questions.

## Supplementary Material

Supplementary figures S1–S8 and table S1–S10 are available at *Genome Biology and Evolution* online (http://www.gbe.oxfordjournals.org/).

Supplementary Data
