# Supplementary material for: Exploring the Limits for Reduction of Plastid Genomes: A Case Study of the Mycoheterotrophic Orchids Epipogium aphyllum and Epipogium roseum
Source: Genome Biol Evol. 2015 Jan 28;7(4):1179–91. doi: 10.1093/gbe/evv019 (PMC4419786; doi:10.1093/gbe/evv019)
Supplement: Supplementary Data [file supp_7_4_1179__index.html]

Exploring the Limits for Reduction of Plastid Genomes: A Case Study of the Mycoheterotrophic Orchids Epipogium aphyllum and Epipogium roseum — Supplementary Data 

# Exploring the Limits for Reduction of Plastid Genomes: A Case Study of the Mycoheterotrophic Orchids *Epipogium aphyllum* and *Epipogium roseum*

## Supplementary Data

files

**Files in this Data Supplement:**

- Supplementary Data - pdf file
- Supplementary Data - xlsx file
