# Supplementary material for: Exploring the Limits for Reduction of Plastid Genomes: A Case Study of the Mycoheterotrophic Orchids Epipogium aphyllum and Epipogium roseum
Source: Genome Biol Evol. 2015 Jan 28;7(4):1179–91. doi: 10.1093/gbe/evv019 (PMC4419786; doi:10.1093/gbe/evv019)
Supplement: Supplementary Data [file supp_evv019_Epipogium_Figure_suppl_new.pdf]

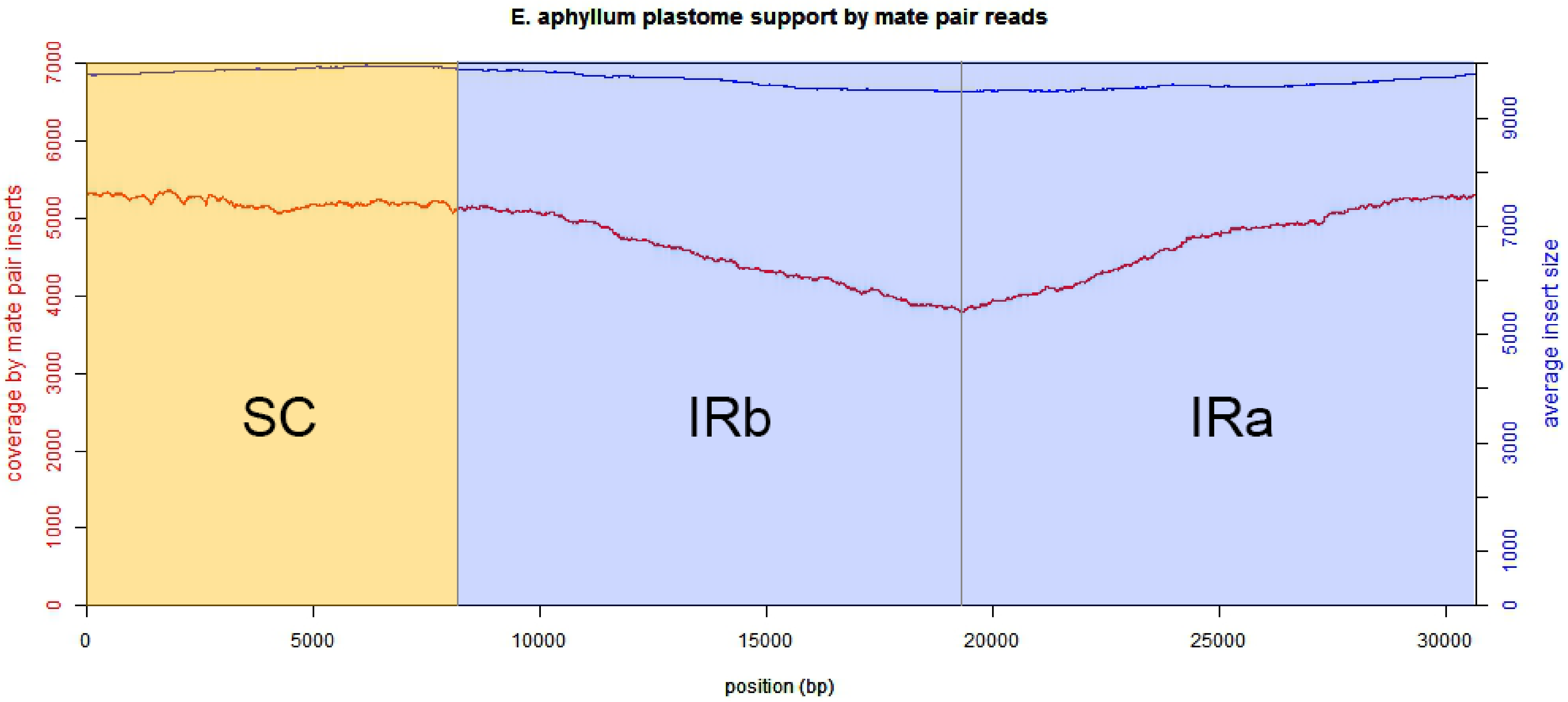

Supplementary figure 1. Mate pair support for the *E. aphyllum* plastome. Coverage by mate pair inserts and average insert size are colored by red and blue, respectively.

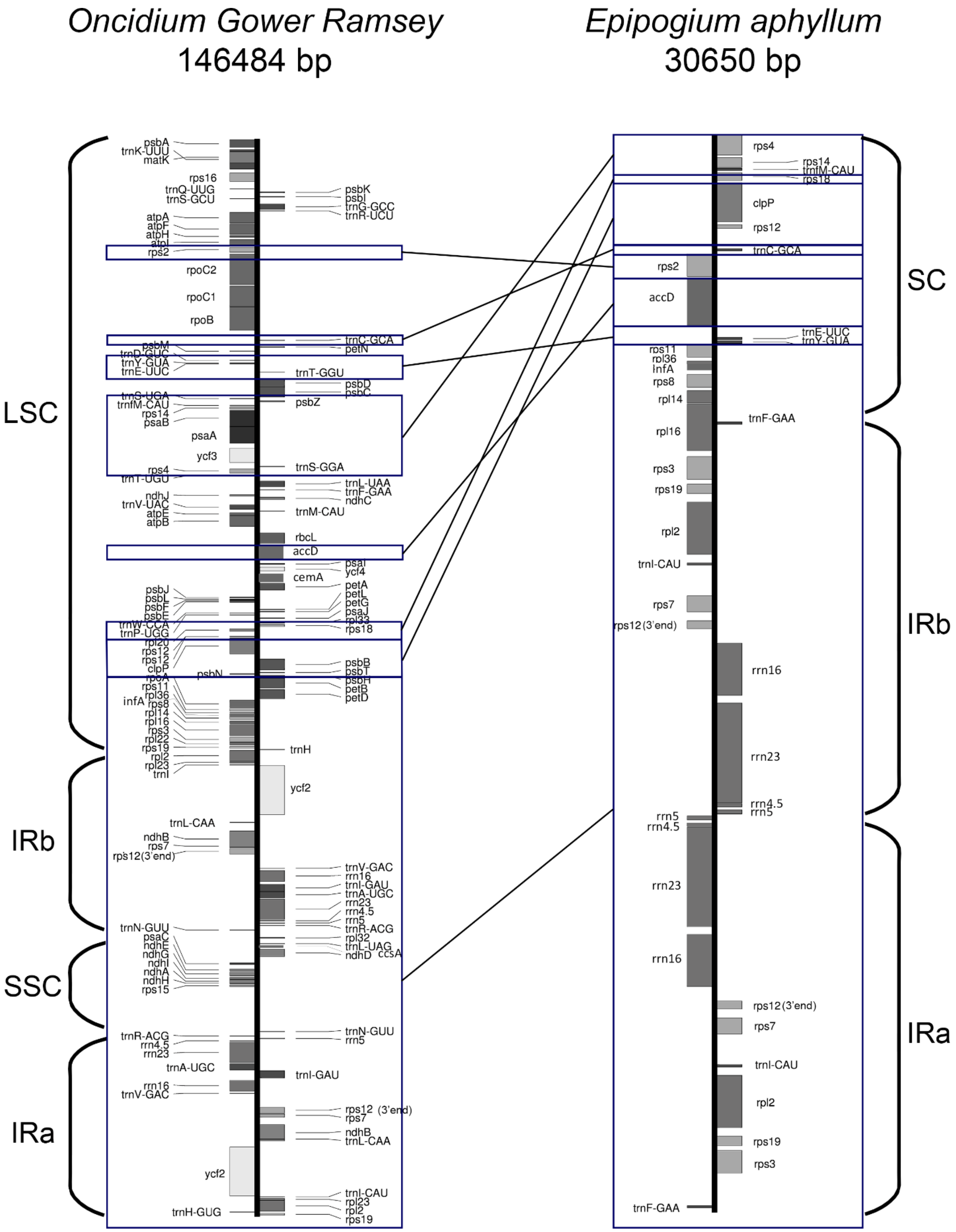

Supplementary figure 2. Collinear blocks between *E. aphyllum* and *Oncidium* Gower Ramsey. The blocks are denoted by blue boxes and the accordance of blocks between species by black lines. Plastome regions (i.e. Inverted Repeat, Single Copy etc.) are indicated. The length is provided for the *E. aphyllum* sample White Sea.

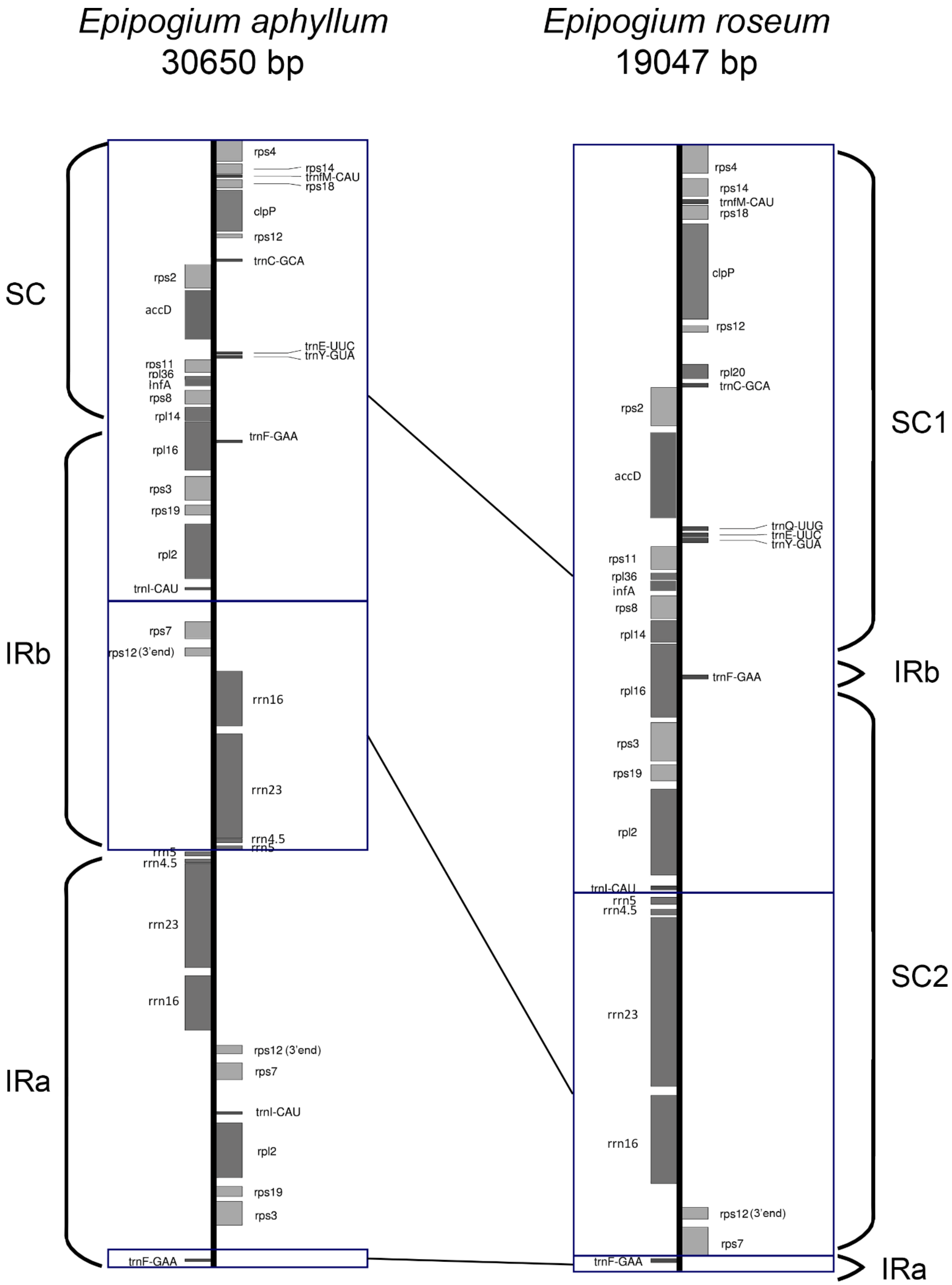

Supplementary figure 3. Collinear blocks between *E. aphyllum* and *E. roseum*. The blocks are denoted by blue boxes, and accordance of blocks between species by black lines. Plastome regions (i.e. Inverted Repeat, Small Single Copy etc.) are indicated. Lengths are provided for the *E. aphyllum* sample White Sea and *E. roseum* sample Vietnam 2.

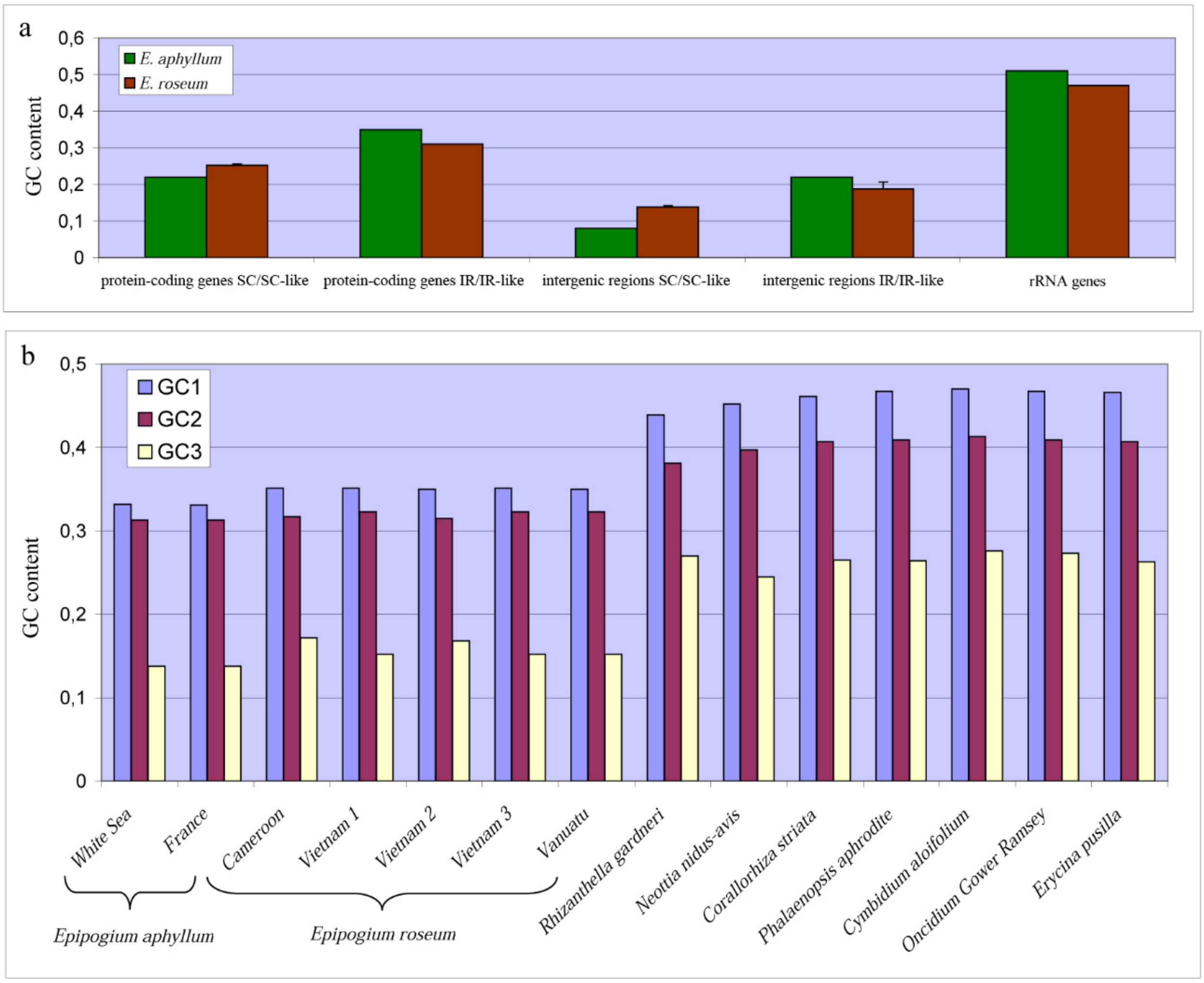

Supplementary figure 4. a: Comparison of GC-content between different regions of *Epipogium* plastomes. "IR-like" refers to a region of *E. roseum* that is homologous to the inverted repeat region of *E. aphyllum*, "SC- like" - aregion of *E. roseum* homologous to single copy region of *E. aphyllum*. Note that although *E. roseum* lost most of its IR, the region homologous to the IR still retains a high GC content compared to the SC-type region. b: GC content in the first, second and third codon positions. The analysis was conducted using 15 common protein-coding genes of orchid species.

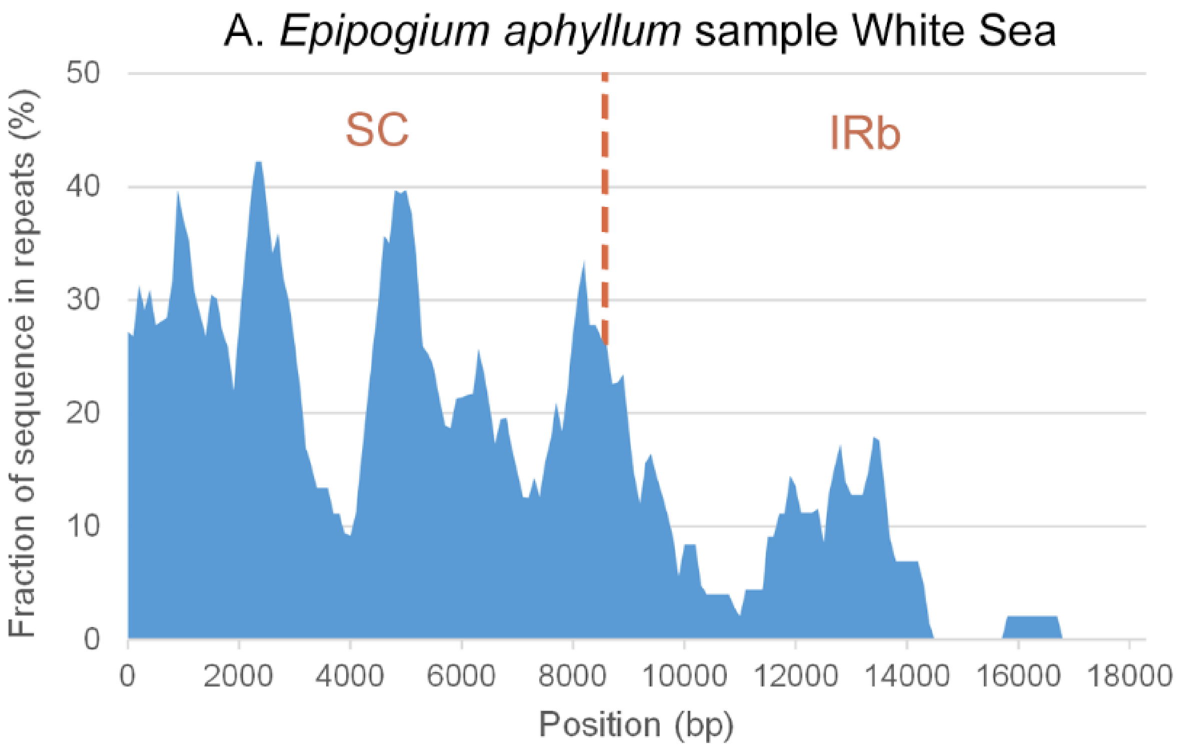

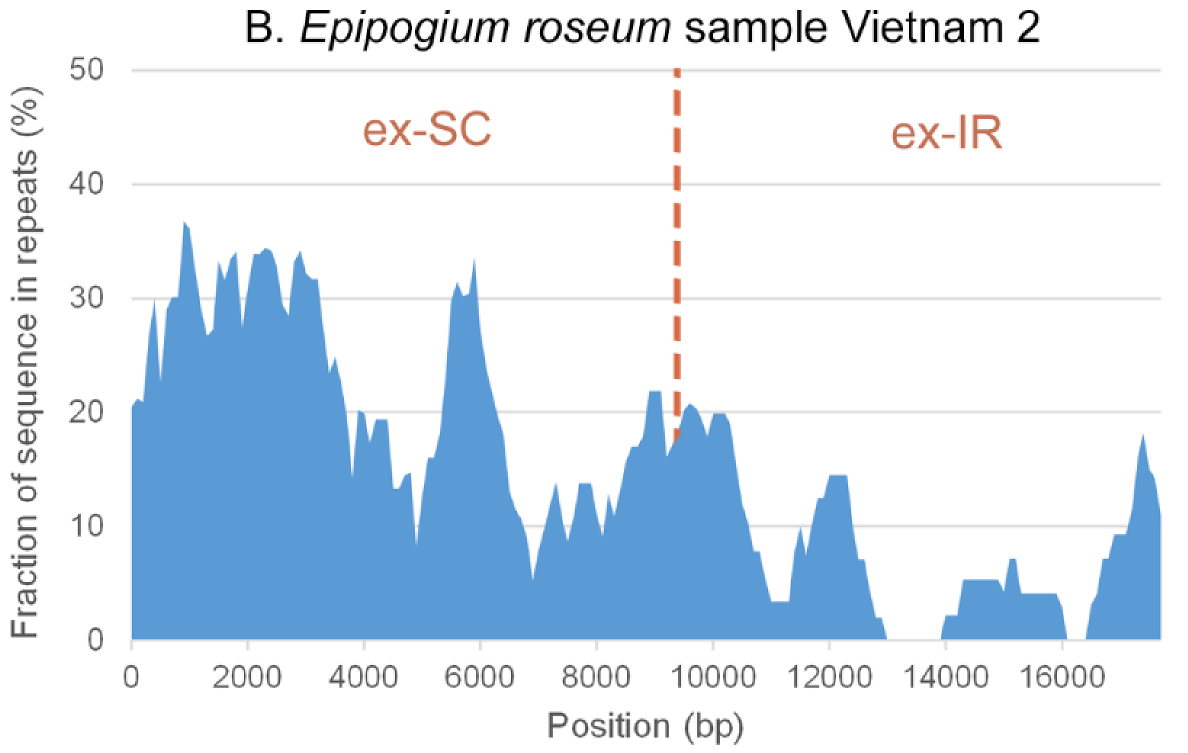

Supplementary figure 5. Repeat distribution in *Epipogium* plastomes. Ex-SC and ex-IR are *E. roseum* regions homologous to E. aphyllum *SC* and *IR*. Only one copy of *E. aphyllum* IR was used for the repeat analysis. The window size for averaging of repeat density is 1,000 bp. The graphs provided here are those for the *E. aphyllum* sample White Sea and *E. roseum* sample Vietnam 2.

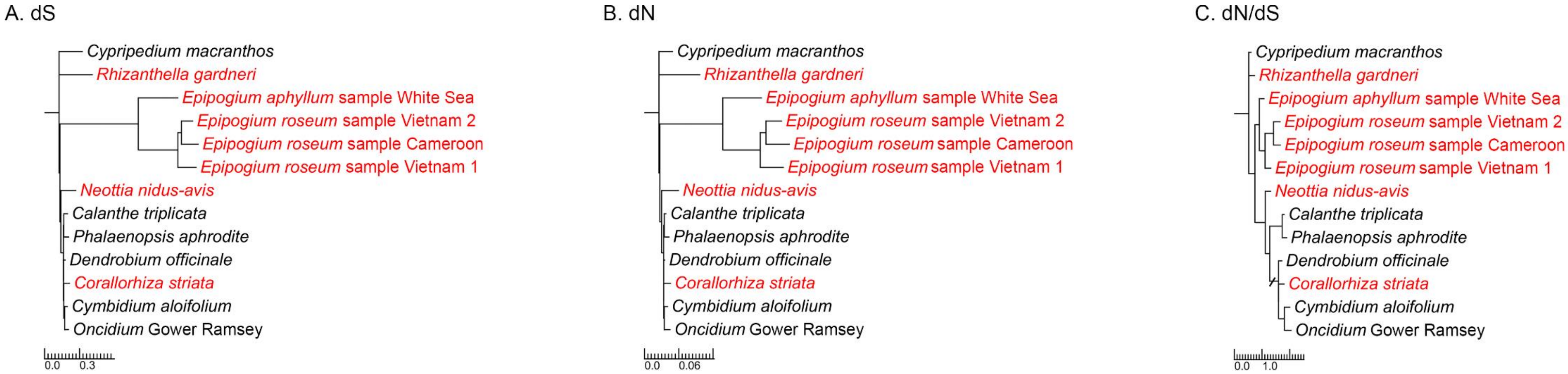

Supplementary figure 6. dS, dN and dNdS in *Epipogium* by branch model. Non-photosynthetic plants are indicated in red. Scale bars denote the values of dS, dN and dN/dS.

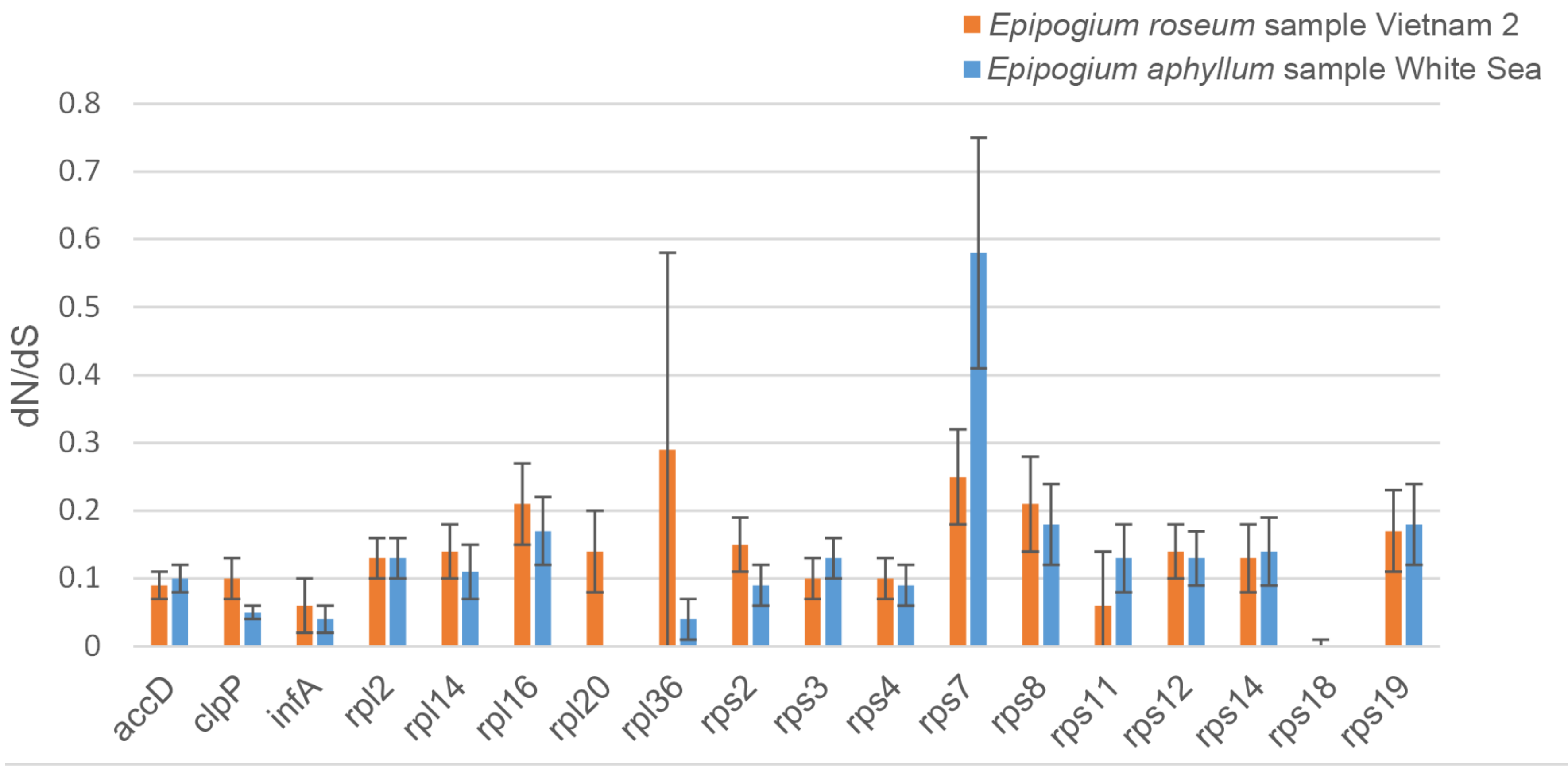

Supplementary figure 7. dN/dS between *Epipogium* and *Oncidium* Gower Ramsey genes. Whiskers show standard errors as estimated by PAML. *rpl20* is absent in *E. aphyllum*.

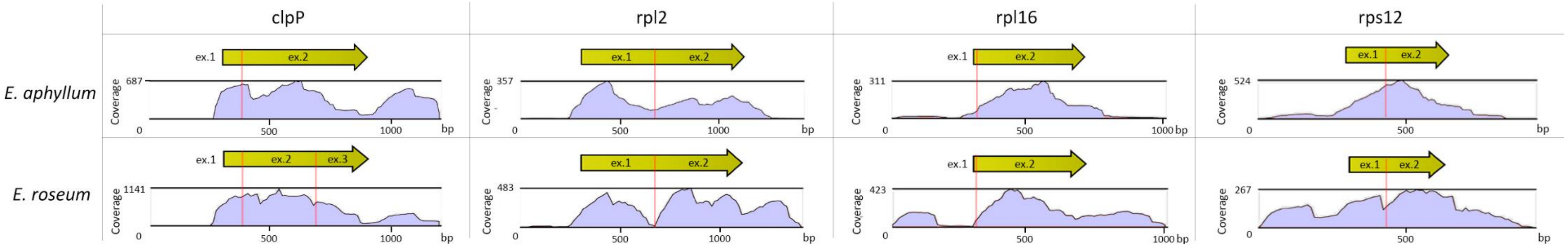

Supplementary figure 8. Splicing verification. Mapping of transcriptome reads onto artificially joined exons of 4 intron-containing *Epipogium* genes and their 300 bp adjacent regions. Filled blue shapes represent read coverage (y-coordinate). The X-coordinate is the position in base pairs. Vertical red lines denote exon junctions. Exons are indicated as “ex.”.
